# Age-related change in flicker thresholds with rod- and cone-enhanced stimuli

**DOI:** 10.1371/journal.pone.0232784

**Published:** 2020-07-08

**Authors:** Amithavikram R. Hathibelagal, Shrikant R. Bharadwaj, Anil R. Yadav, Ahalya Subramanian, James R. E. Sadler, John L. Barbur

**Affiliations:** 1 Brien Holden Institute of Optometry and Vision Science, L V Prasad Eye Institute, Hyderabad, India; 2 Brien Holden Eye Research Centre, L V Prasad Eye Institute, Hyderabad, India; 3 Centre for Applied Vision Research, School of Health Sciences, City, University of London, London, England, United Kingdom; 4 Human Performance, QinetiQ, Hampshire, England, United Kingdom; University of Massachusetts Medical School, UNITED STATES

## Abstract

**Purpose:**

Rod and cone photoreceptor-specific tests can be time-consuming. A new non-invasive test is described. The test is based on the measurement of flicker modulation thresholds with rod- and cone-enhanced visual stimuli, which requires only minimum adaptation time. Here, we investigated how the rod-and cone-mediated flicker thresholds vary with age.

**Methods:**

Monocular thresholds with rod and cone–enhanced stimuli were measured in 140 healthy adults, (age range: 18–75 years), foveally (0°) and at four parafoveal locations, at an eccentricity of 5° in each of the four quadrants using five, adaptive, interleaved staircases. Temporal frequencies, stimulus sizes, background luminance and spectral composition, were adjusted appropriately to achieve approximately 1 log unit separation in sensitivity between the rod- and cone-enhanced stimuli. Spectrally calibrated, ‘neutral density’ filters were used to enable adequate control of display luminance for rod enhanced stimuli.

**Results:**

The magnitude of central and parafoveal rod thresholds was significantly higher than the central and parafoveal cone thresholds, respectively (p < 0.001) in both the age groups. However, the rate of increase in central rod thresholds (y = 0.45x—12.79; linear regression equation) was not significantly steeper than the rate of increase in central (y = 0.29x—8.53) cone thresholds (p = 0.15). Centrally, cone thresholds showed a better correlation with rod central thresholds for the age > 45 years (Spearman correlation, ρ = 0.74, p < 0.001) compared to age ≤ 45 years (ρ = 0.41, p < 0.001).

**Conclusions:**

Thresholds with rod- and cone-enhanced stimuli are largely invariant below 45 years of age and increase rapidly above this age. This age-wise normative database can be used as an effective functional-marker to assess photoreceptor sensitivities in retinal diseases.

## Introduction

Flicker Modulation Thresholds (FMT) can provide a sensitive measure of changes in the temporal responses of the visual system [1]. Flickering stimuli generate trains of neural modulation and this load accounts for an increased demand on the blood supply [2–5] and overall metabolic activity in the retina [6], when compared to static stimuli. Flicker modulation thresholds can, therefore, act as a useful photoreceptor-specific functional biomarker in early detection of diseases such as Age related Macular Degeneration (AMD) [7] and Diabetic Retinopathy [8–10], where hypoxia plays an important role in disease pathogenesis [11–18]. For the same reason, these thresholds can also be useful in assessing the severity of rod and cone dysfunction [7]. For instance, flicker modulation thresholds measured at a single temporal frequency such as at 14 Hz is considered to be a sensitive indicator of cone function in patients with AMD, with regards to ease of testing and repeatability [7, 19]. It has also been shown that the differences in cone FMT between age-matched healthy individuals and patients with AMD are largest in the central 5° of fixation [17], which is within the region of maximum vulnerability to damage in these patients [20, 21]. The absolute rod detection threshold, which is typically measured after a period of dark adaptation (20–30 minutes), provides a useful measure of rod function [22–28]. For rod-mediated vision, this test should also overcome the requirement of long dark-adaptation times that make the test results more variable [7] and unattractive for use in a clinical setting. Given the previous literature, it is desirable to develop an efficient test to measure rod- and cone-specific sensitivities within the central vision and to account for the effects of normal, healthy ageing. Such data are needed to detect loss of sensitivity that falls outside the normal limits as one might expect in patients at risk of developing and quantify the earliest losses in patients with AMD or diabetes.

It is well established that in normal, healthy ageing, photopic flicker modulation thresholds increase with increasing age [29–38], although this increase only starts to become significant beyond 40 to 45 years of age [29, 30]. An earlier study reported little or no change in absolute scotopic thresholds with age [39]. However, the small sample size (n = 23) and the maximum age of the participants in that study (the oldest subject was 61 years of age) [39], might have precluded the authors from establishing the true variation of scotopic thresholds with age. Subsequent studies reported greater increases in scotopic thresholds with increasing age [40–43]. In particular, Jackson et al. found that the rate of age-related increase of scotopic thresholds (-0.08 log units/decade) is faster than that measured for photopic thresholds (-0.04 log units/decade) [40]. However, it is important to note that the previous studies tested absolute scotopic thresholds using a static target after a prolonged period of dark adaptation (20–30 minutes). In the current study, we have employed a rod / cone flicker test, which makes use of stimulus parameters such as background luminance, spectral composition, temporal modulation frequency and stimulus size to test for rod—and cone—specific responses at five discrete locations in central vision (5°), with minimal adaptation time. The findings from this study characterize the existing variability within rod- and cone-mediated thresholds in central and near peripheral vision and establish the upper, threshold limits one can expect in healthy, normal ageing (20–75 years). The latter can be used to detect when rod- or cone-specific thresholds exceed normal age-matched limits as a result of subclinical disease. In addition, the rod / cone flicker modulation threshold test can also be used to monitor the progress of a disease or the effectiveness of treatment.

## Materials and methods

### Participants

The study participants had normal vision with high-contrast logMAR visual acuity (VA) equal to better than 0.0 (20/20 Snellen fraction) and no history of ocular and systemic disease. Participants were recruited from the staff and student cohort at the L V Prasad Eye Institute (LVPEI), Hyderabad, India and from those patients who visited the institute for a regular eye examination, with no specific complaints. All the participants were of Indian ethnicity. The participants underwent ocular examination including, slit lamp biomicroscopy and fundus examination to rule out any ocular pathology. Intraocular pressure was measured using Goldmann applanation tonometry and was also within normal limits. Participants with any ophthalmic pathology or with lens status (nuclear, cortical or posterior subcapsular) graded as LOCS III, or higher were excluded [44]. In addition, participants who were unable to complete successfully the ‘learning’ mode of the test were also excluded. The ethics and protocol of the study was approved by the Institutional Review Board of LVPEI and all the procedures were conducted in accordance with the tenets of the Declaration of Helsinki. Written informed consent was obtained from all the participants before they took part in the study. A sample of approximately 150 participants could be considered as a representative of the population to derive a normative database for visual functions [45].

### Flicker-*Plus*

**Fig 1:**
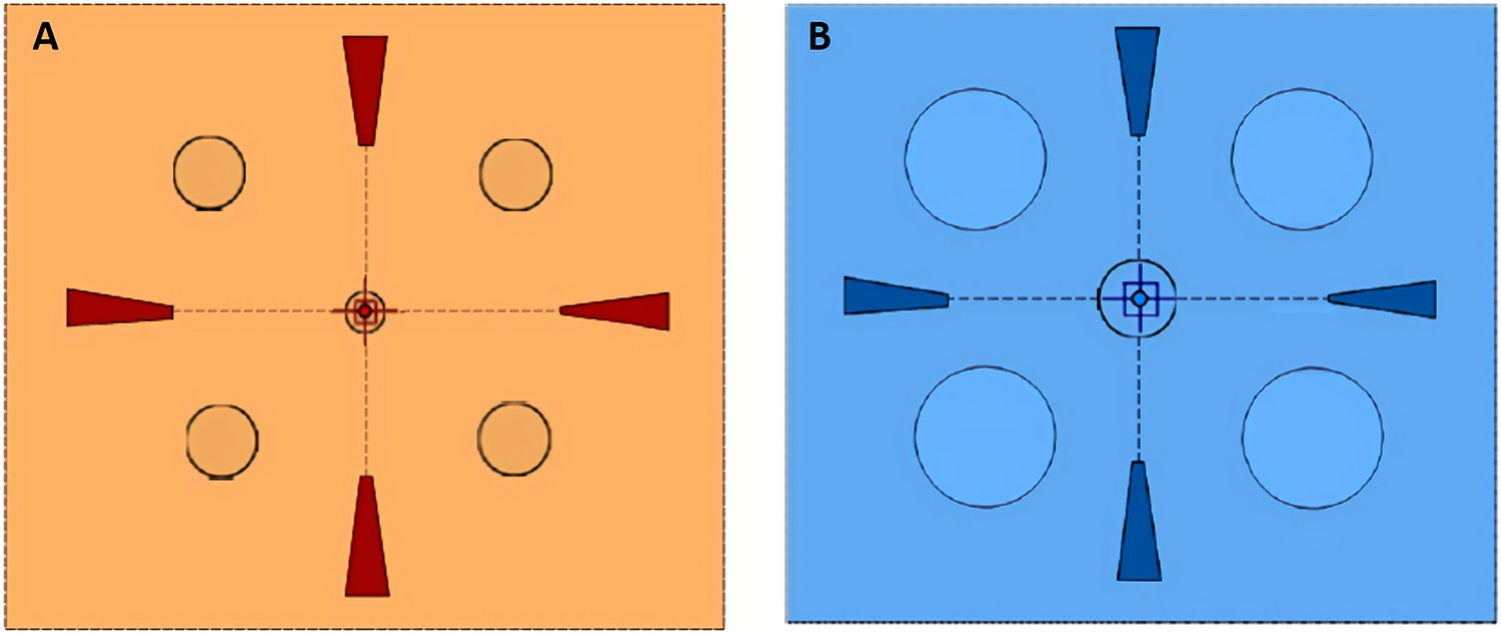
Background display for cone and rod-enhanced stimulus conditions. The left panel shows the approximate appearance of the visual display when showing the cone-enhanced stimulus. The stimulus is displayed either at the centre or at an eccentricity of 5° (away from fixation), diagonally in each of the four quadrants. The real stimulus is a borderless disc, modulated sinusoidally and presented at equal luminance. The right panel illustrates the appearance of the rod-enhanced stimulus condition. The subject views the display through a 1.0 log unit neutral density filter to ensure reliable display performance at 0.5 cd/m^2^. Preliminary experiments show that the measured rod thresholds do not change significantly when the time for background adaptation is extended from 1 to 16 minutes.

Flicker modulation thresholds (FMTs) were measured using the Flicker-*plus* module of the commercially available Advanced Vision and Optometric Tests (AVOT) developed at City, University of London [30]. The AVOT tests run on a desktop computer with two displays, one for the experimenter and the other for generating the visual stimuli for a number of advanced vision tests. The display used by the experimenter to run the tests and the calibrated stimulus display viewed by the participant are separated by a black curtain, such that the participant can only see the stimulus display. The display monitor specifications were as follows: 24" IPS (in-plane switching) LCD monitor (EIZO, Model ColorEdge CS2420; EIZO corporation, Japan) using a 10-bit graphics card with 1920 X 1200 resolution at a frame rate of 60 Hz. The calibration of the monitor is performed using a photometer (Mavo-Monitor USB, Gossen, Germany) and a custom-built software (LUMCAL; City Occupational, Ltd., London, UK). The stimulus was a uniform disc of appropriate size (see Table 1 for details) and was presented either at the centre (0°) of the display (as indicated by the four guides shown in Fig 1), or in one of the four quadrants (5° eccentricity from fixation) namely supero-temporal (ST), supero-nasal (SN), and inferonasal (IN) and inferotemporal (IT) quadrant, depending on the eye that was tested (Fig 1). The duration, temporal frequency, background luminance and spectral power distribution of the background and target were selected to produce either cone [30]- or rod-enhanced stimuli (Table 1). The chromaticities and other parameters were different between rod and cone—enhanced stimuli (Table 1). However, in each of these conditions, the target and background had the same spectral composition. The differences in the spatiotemporal properties between rod and cones were exploited in this test such that the rod-enhanced condition had stimuli with relatively longer display duration, lower temporal frequency and light levels, all relative to the cone-enhanced conditions [46–49]. The stimulus parameters for the two conditions were selected to produce approximately the same flicker thresholds for both rods and cones. Preliminary results in CNG-B3 patient with retinal achromatopsia confirm that the cone-enhanced thresholds can still be detected by rods, but with thresholds approaching the maximum that can be generated on the visual display (currently in preparation). Five, randomly interleaved 2-down 1-up adaptive staircases, one for each stimulus location [30], were used to measure the corresponding FMTs. During each test, the five interleaved staircases determine either the subject’s rod- or cone-mediated thresholds. Each threshold was estimated by averaging the last six reversals. The implementation of the 5AFC staircase with variable step sizes and the 2-down / 1-up approach reduces the chance probability of a correct response to 1/25. The measured thresholds correspond to just over 70% probability of a correct response [50]. The participant viewed the stimulus display monocularly from 1m. Only one eye (dominant eye or eye with a visual acuity of at least 0.0 logMAR or better) per participant was tested.

**Table 1:** Stimulus parameters used for cone—and rod—enhanced stimulus test conditions.

Stimulus parameters	Cone–enhanced	Rod–enhanced
Temporal frequency (Hz)	15	5
Size of stimulus (min of arc)	30’–centre and 60’—periphery	45’–centre and 90’–periphery
Chromaticity co-ordinates (x, y)	0.58, 0.46	0.18, 0.089
Scotopic/photopic ratio*	0.7	8
Luminance (cd/m^2^)	24	0.5
Duration (ms)	334	600

* The ratio of the scotopic to the photopic luminance of the stimulus is often known as S/P [51].

### Procedure

The Flicker-*Plus* test includes a ‘learning’ option which is used to familiarize the participant with the bespoke, numeric keypad buttons needed to indicate the position of the stimulus on display during the test procedure. The test stimulus was always preceded by a briefly presented fixation stimulus at the centre of the screen. The latter consisted of a dark square outline and a cross, designed to attract the participant’s point of regard. The participant was instructed to maintain their fixation on the location of this marker throughout the test to ensure that the peripherally presented stimuli were seen at 5° eccentricity. The raised, keypad buttons mirrored the five locations of the stimulus on display to make the task easier. Each participant was expected to achieve 100% correct responses in the learning mode before the full test was administered. The order of the rod and cone flicker testing was randomized. The rod-enhanced condition required the participant to adapt to the background field for 1½ minutes, wearing spectrally-calibrated, 1.0 log unit neutral density filter (Oakley Half Jacket 2.0 –Black Iridium, USA). Each test run took ~10 minutes to complete and breaks were rarely required. Pupil size was not measured during the experiment as the flicker modulation at equal luminance remains largely independent of pupil size. Previous research has also shown that small pupil size changes have only minimal effects on FMTs measured at photopic and high mesopic light levels [33]. Pre-retinal filters such as the short-wavelength absorption of light by the lens and the macular pigment do not affect the stimulus modulation depth when the spectral power distribution of the test remains unchanged and the same as that of the adjacent uniform background [52].

### Data analysis

The data were not normally distributed as tested by the D’Agostino & Pearson test (p <0.05); therefore non-parametric statistical analyses were carried out. The age of 45 years was chosen as the demarcation point between the two groups based on previous literature [29] for further analyses. We have examined the results using the point of separation as a free parameter in order to determine whether there was a justification for using a fixed cut-off point at 45 years. We examined all test conditions and found that the mean transition age was 46.5 ± 1.0 years. In view of this observation, the data were fitted with two separate linear functions for age group ≤ 45 years and for group > 45 years using a linear regression model in GraphPad Prism (Graph Pad Software, Inc., CA, USA). Spearman correlation coefficient was computed to analyze how FMTs vary as a function of age separately for the two age groups (≤ 45 years and > 45 years).

## Results

**Fig 2:**
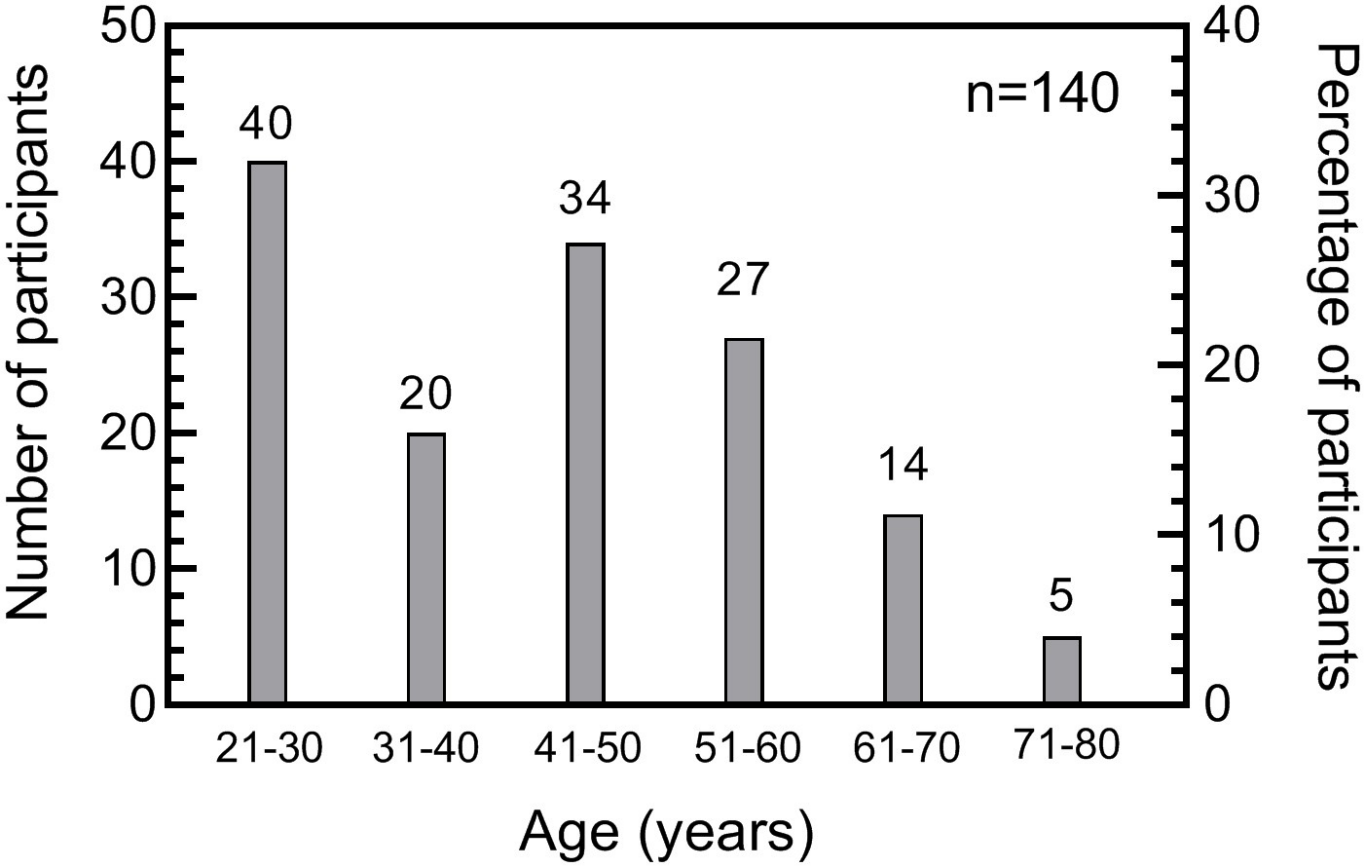
The number of participants across all age groups, who participated in the experiment. The numbers on top of the bars indicate the number of participants in that group.

As mentioned in the methods, we aimed to recruit 150 subjects. However, we ended up recruiting a total of 145 participants (82 males and 63 females). Out of 145, five participants were excluded, as they had difficulties with the ‘learning mode’ and were unable to either see the flickering stimulus or to complete the test. Therefore, a total of 140 participants completed the study. The mean age (SD) of these participants was 41.81 (15.11) years. Nearly 4% (5/140) of the study participants were pseudophakic in the tested eye. The number of participants across each decade who participated in the study is shown in Fig 2. Mann -Whitney test showed no significant differences between male and female groups for central (p = 0.80) and parafoveal cone FMT (p = 0.17). Similarly, Mann -Whitney test showed no significant differences between male and female groups for central (p = 0.40) and parafoveal rod FMT (p = 0.78).

**Fig 3:**
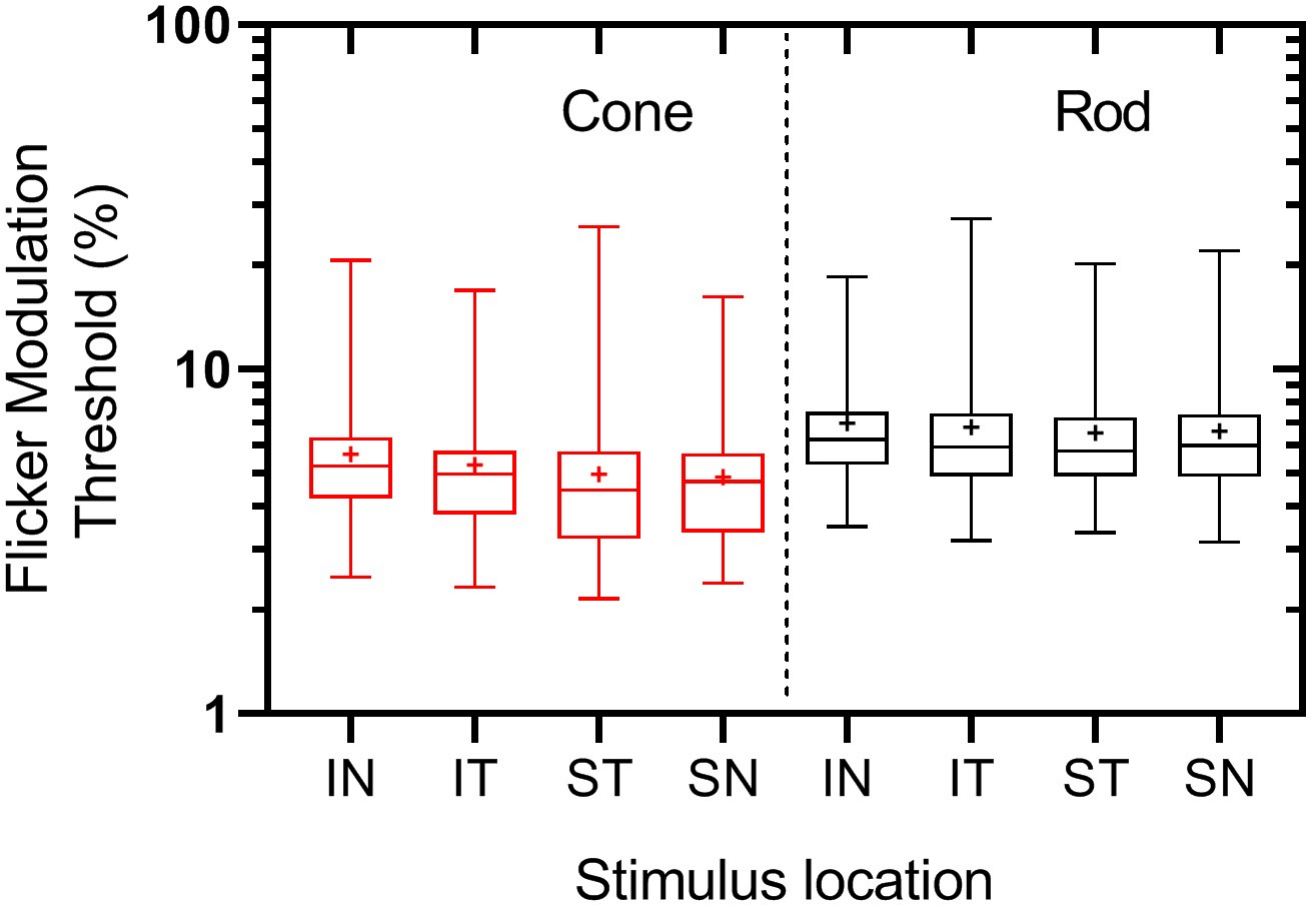
Box plot of cone and rod (FMT) obtained in the four parafoveal locations (inferonasal—IN, inferotemporal -IT, superotemporal—ST, superonasal- SN) across all participants. Box plot showing the median, first quartile, third quartile, minimum and maximum FMT for each test location. Note that rod thresholds are slightly higher than cone thresholds. The plus symbol indicates the mean of each group.

**Fig 4:**
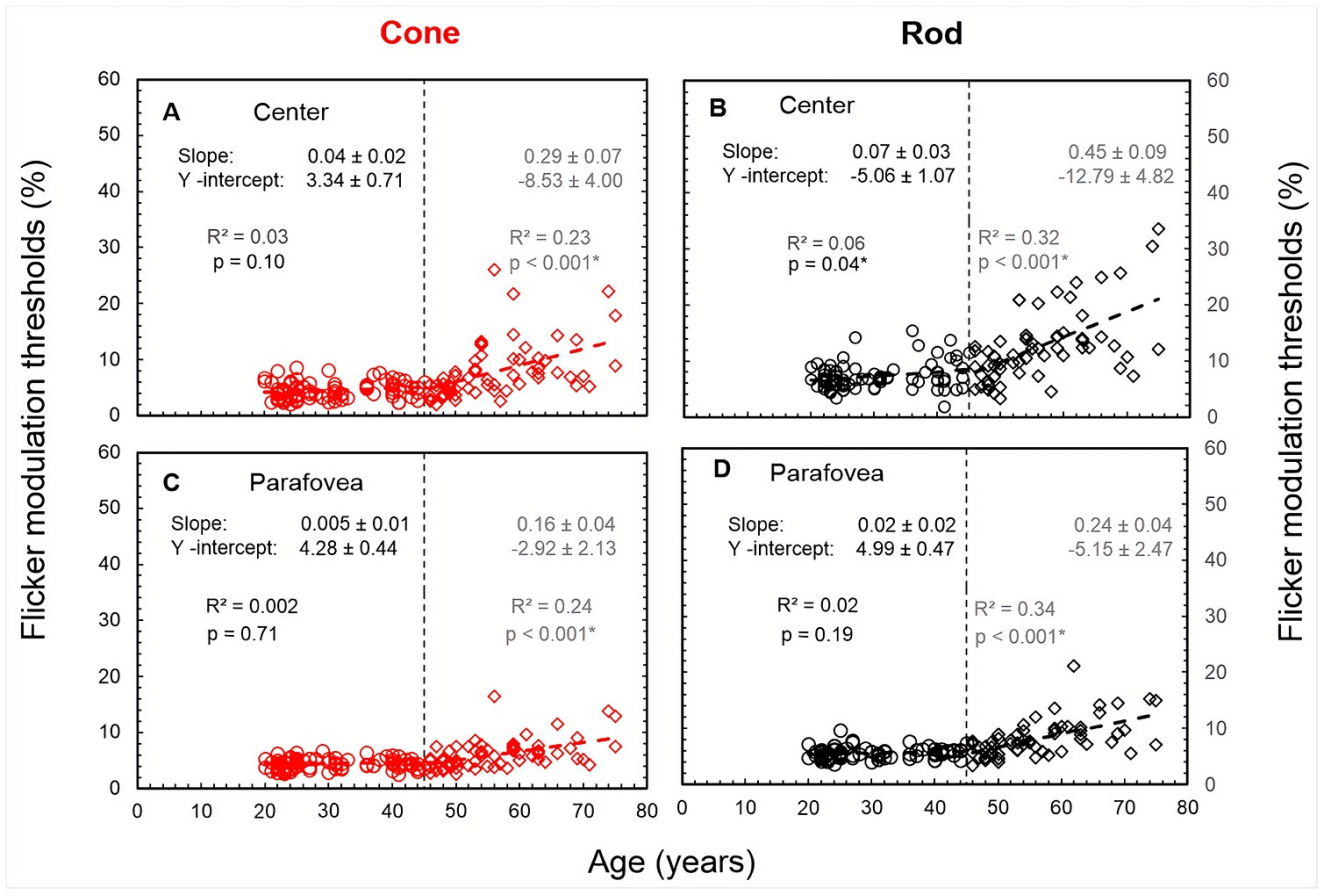
Cone (left panels) and rod (right panels) FMT as a function of age for central and parafoveal locations. Linear regression analysis based on least squares method was performed separately on both segments (≤ 45 years and > 45 years) to obtain the best fit. The dotted, vertical lines indicate the chosen cut-off age, which is 45 years. The slopes (± SE, standard error) and the y-intercept (SE) is denoted separately for both age groups in each of the panels. The variance of FMTs that can be expressed by variation in age is indicated by the R^2^ values, adjusted for the sample size. The asterisk symbol in the equations indicated if the slope was significantly different from zero.

Fig 3 revealed no noticeable differences across the four parafoveal locations for cone thresholds and well as for rod thresholds. Therefore, the cone and rod thresholds in the four parafoveal locations were averaged separately, and only the mean values were used for each participant. The slopes of the regression lines between FMT and age were compared to determine if they were different from zero (i.e. if there was an age-related trend in the data) and if they were different from each other (i.e. if the rate of change in FMT’s were different across different test conditions). A p-value of ≤ 0.05 was the requirement for statistical significance. Except for cone thresholds (central and parafoveal) in the younger age group (Fig 4A and 4C) and parafoveal rod thresholds in the younger age group (Fig 4D), all the other slopes were significantly different from zero (p < 0.05). The rate of increase in central cone thresholds (i.e., 2.9% per decade–Fig 4A) above 45 years of age is not significantly different to the rate of increase in central rod thresholds (i.e., 4.5% per decade—Fig 4B; p = 0.15; See Table 2) for the same age group. The increase in parafoveal cone thresholds (i.e., 1.6% per decade–Fig 4C) above 45 years is also not significantly different than the measured increase in parafoveal rod thresholds (i.e., 2.4% per decade–Fig 4D; p = 0.19; See Table 2) for the same age group.

**Table 2:** Median and IQR for rod and cone-mediated thresholds for two age groups, and Spearman correlation between thresholds and age.

			Thresholds (%)				
Location	Age group	Condition	Median	IQR^#^	Slopes^		Spearman correlation(ρ)
Centre	≤ 45 years	Cone	4.24	2.00	0.03	p = 0.30	0.22
	(n = 79)	Rod	6.80	2.60	0.07*		0.19
	> 45 years	Cone	6.72	5.29	0.29*	p = 0.15	0.60*
	(n = 61)	Rod	11.75	5.65	0.45*		0.56*
Parafovea	≤ 45 years	Cone	4.43	1.54	0.005	p = 0.47	0.069
	(n = 79)	Rod	5.48	1.27	0.02		0.24
	> 45 years	Cone	5.76	3.04	0.16*	p = 0.19	0.50*
	(n = 61)	Rod	7.48	3.84	0.24*		0.61*

* The correlations and slopes (different from zero) that are significant for a p—value significance set as 0.05.

^#^ (75^th^ percentile-25^th^ percentile).

^ The slope values were obtained from linear regression between thresholds and age.

**Fig 5:**
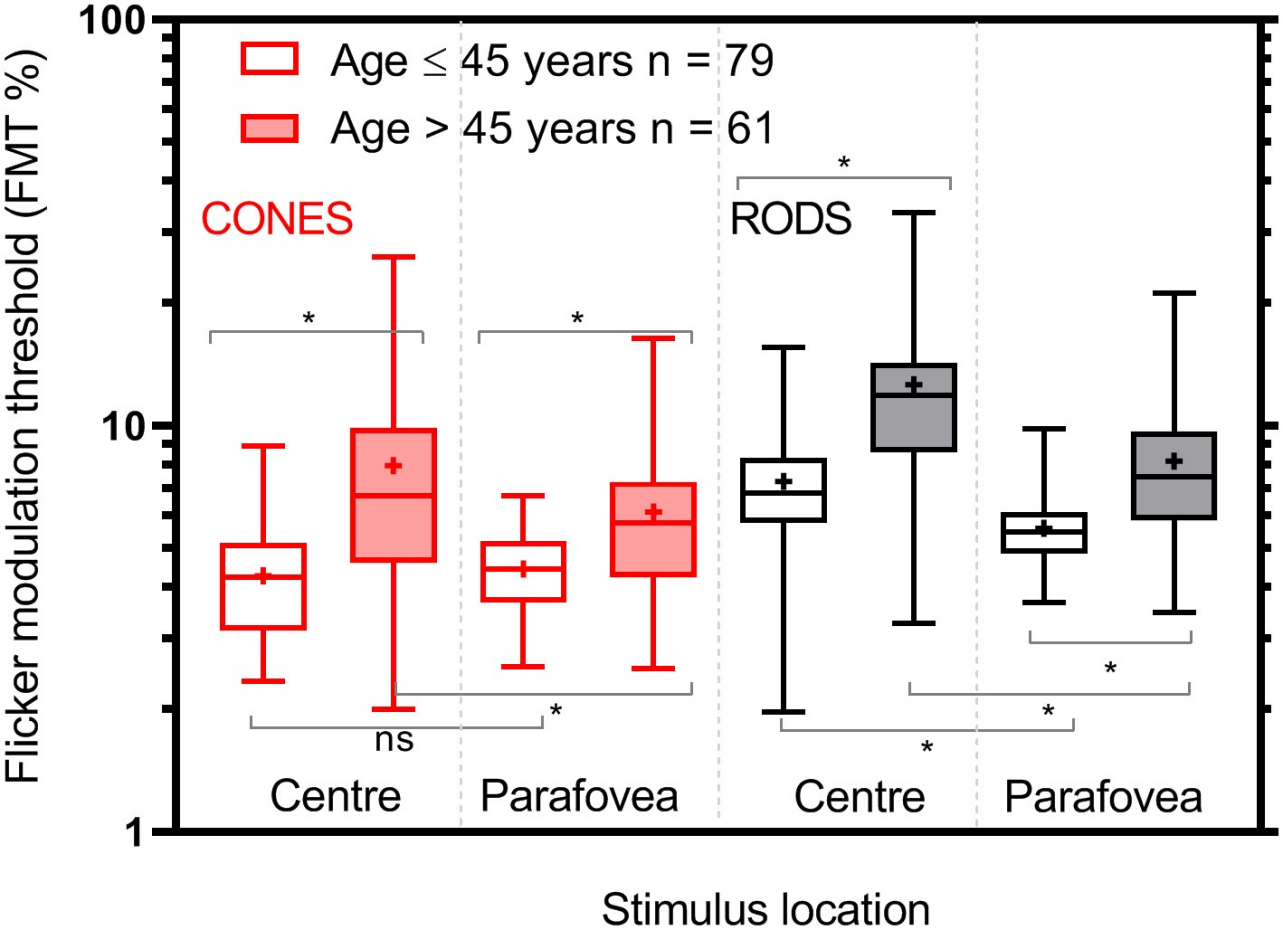
Box plots of cone enhanced and rod enhanced FMTs at the centre and parafoveal locations for both age groups. Box plot showing the median, first quartile, third quartile, minimum and maximum FMT for each cohort. The solid boxes represent younger age cohort (< 50 years), and unfilled boxes represent elderly cohort (≥ 50 years). The plus symbols refer to the mean of each distribution. The asterisk indicates the comparisons that were significantly different from each other.

The slopes and the y-intercepts of the regression lines were also compared separately as a function of stimulus location (between centre and parafovea) in cone and rod conditions. In the younger age group (≤ 45 years) cone threshold slopes and y-intercepts between the centre (0.4% per decade) and parafovea thresholds (0.05% per decade) were not significantly different from each other (p = 0.24 and p = 0.36 respectively). In the elder age group (> 45 years), there are no significant differences in the slopes for the cone thresholds between central (2.9% per decade) and parafoveal locations (1.6% per decade) (p = 0.10). There was, however, a significant difference between the y—intercepts of central and parafoveal cone FMT (p = 0.004; Fig 5) in the age group > 45 years. Similarly, in the younger age group, the slope for rod thresholds in centre (0.7% per decade) was not significantly different from the rod thresholds measured at the parafoveal locations (0.2% per decade) (p = 0.15). The y-intercepts were, however, significantly different for rod thresholds between central and parafoveal locations (p < 0.001) in the younger age group. In the elder age group, the rate of change in central rod thresholds (4.5% per decade) was significantly greater (p = 0.03) than that measured in parafoveal vision (2.4% per decade). The y-intercepts could not be determined as the slopes differed significantly.

**Fig 6:**
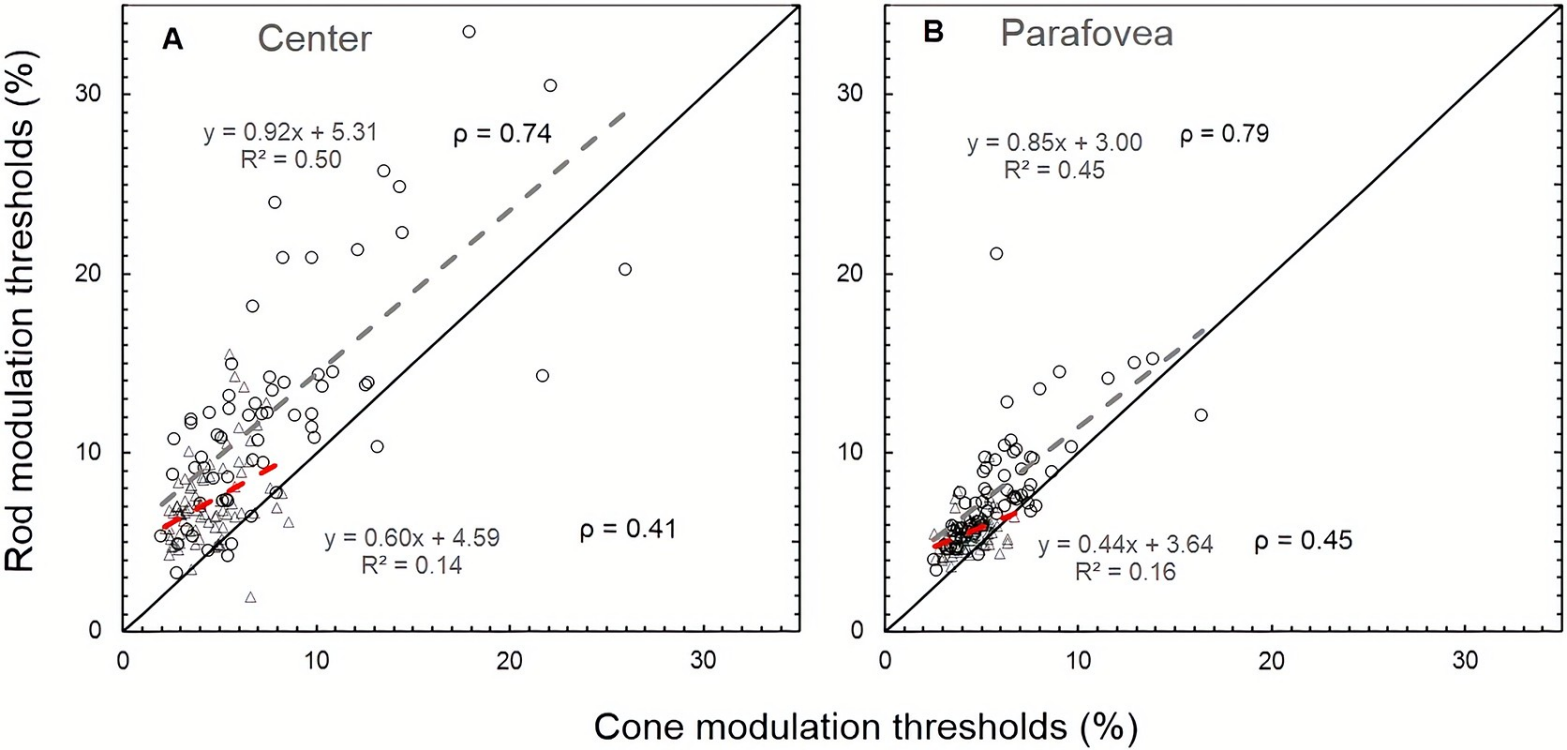
Central cone thresholds are plotted against corresponding central rod FMTs (panel A). Parafoveal cone thresholds are plotted against corresponding parafoveal FMTs (panel B). The data are plotted separately for two age groups (≤ 45 years and > 45 years) in each of the panels. The solid straight line in both the panels indicates the line of equality. The open triangle symbols and dashed (red) line indicate the data and linear fit, respectively for younger age data (≤ 45 years). The open circles and dashed (grey) line indicate the linear fit respectively for group > 45 years in each of the panels. The Spearman correlations (ρ) between cone and rod thresholds are also indicated in the figure, and all the correlations are statistically significant (p < 0.001).

There is no significant correlation between flicker (rod and cone) thresholds and age up to the age of 45 years. However, beyond 45 years of age, there is significant correlation for both rod and cone FMTs with age (p < 0.05) as shown in Table 2. Approximately 89% (125/140) of the participants showed higher rod FMTs compared to cone FMTs both centrally (Fig 6A) and parafoveally (Fig 6B). The linear regression was performed between central cone and rod thresholds (central) for both age groups (≤ 45 years and > 45 years) separately. The slopes (≤ 45 years: y = 0.60x+4.59, R^2^ = 0.14, > 45 years: y = 0.92x + 5.31, R^2^ = 0.50; p = 0.12) were not significantly different from each other (Fig 6A). However, parafoveally slopes were significantly different from each other (≤ 45 years: y = 0.44x+3.64, R^2^ = 0.16; > 45 years: y = 0.85x+3.00; R^2^ = 0.45; p = 0.007).

## Discussion

This study reports measured flicker modulation thresholds as a function of age at five locations in central vision with stimulus conditions that favour either rods or cones. The new test employed in the study benefits from minimum times of adaption to the background luminance and the use of a statistically-efficient, 5-alternative, forced choice procedure to measure flicker modulation thresholds that are not affected significantly by residual refractive errors, higher order aberrations or moderate levels of scattered light The results show almost constant rod- and cone-mediated flicker sensitivities up to 45 years of age and a more rapid decline above this age (Fig 4). The age and stimulus location dependence of rod and cone data is discussed separately in the following sections.

### Cone flicker thresholds as a function of age and test location

Our findings show that 15Hz cone FMTs measured at the point of regard remain mostly unchanged up to 45 years, followed by a steeper increase above this age (Table 2). This finding is in agreement with earlier findings, which suggest that the variation of foveal flicker thresholds (< 20 Hz) across the life span has three phases namely, the initial decrease in thresholds up to 16 years of age, the stable phase between 16yrs until 45–50 years and an increase beyond 50 years of age [29, 37]. It is well established that ageing causes significant changes in the optics of the eye as well as structural/anatomical changes in the retina, RPE and the choroid [53–55] and neural changes in the post-receptoral pathways. Here we explore the possible factors that contribute towards the increase in cone FMT with increasing age. Firstly, the steady-state pupil size decreases with age [56], and this causes a reduction in retinal illuminance, which in turn can potentially affect FMT [33]. Previous studies have, however, shown that the increase in FMTs in the elderly is higher than predicted by age-related miosis [31, 32]. Besides, the Flicker-*plus* test was designed to ensure that there is no change in the mean luminance level during the stimulus presentation. Both E_b_ (background retinal illuminance) and E_s_ (stimulus modulation retinal illuminance) are both directly proportional to pupil size. Therefore, pupil size affects the corresponding retinal illuminances but does not affect the temporal contrast modulation of the stimulus or FMT (i.e., the measurement variable). Similarly, this finding of increased FMT with ageing is unlikely to be associated with changes in cone density, as the reported decrease in cone numbers in the central ± 5° of the retina is small [57–62]. Factors such as the decrease in ganglion cell density, especially parasol ganglion cells in the magnocellular (MC) pathway with increasing age [53, 55, 63] may be attributed to the increase in FMT. This is supported by a previous finding that in patients with severe parvocellular lesions, contrast thresholds for temporal frequencies (1–32 Hz) is largely mediated by the MC pathway [64]. However, the effect of ageing-related structural changes in neurons in the human post-receptoral pathways is unclear and is limited to animal studies [65]. Previously, in monkeys and cats, it has been shown that cortical neurons’ (V1 area) sensitivity and the signal-to-noise ratio is decreased significantly in older age group compared to the younger age group [66–68]. These results suggest that similar ageing changes in human cortical neurons are conceivable and which could account for an increase in FMT. Besides, the estimated sampling efficiency related to contrast threshold task in older human subjects is significantly poorer than younger subjects [69], which may also indicate a neural basis for loss in contrast sensitivity with increasing age.

Although the results show that the rate of increase in cone thresholds with increasing age was comparable between central and parafoveal locations (p = 0.10), it is important to explore the possible reasons for the rate of changes in cone FMT (Fig 4A and 4C). This finding of steeper slopes for central cone thresholds is in agreement with the results of a recent study, carried out under similar test stimulus conditions, where foveal photopic thresholds had steeper slopes for those over > 40 years of age, compared to parafoveal locations (4°) [30]. Other studies failed to find a significant increase in thresholds in cone-mediated central vision (0°) with increasing age [34, 70]. The differences could be due to different stimulus parameters such as adapting luminance, size, temporal frequency and presentation time. More specifically, in this study, the size of the central flicker stimulus (30’) was smaller compared to the peripheral stimulus (60’) to account for spatial summation [34, 70].

### Rod flicker thresholds as a function of age and test location

In a way similar to cone-mediated thresholds rod thresholds (central and parafoveal) are significantly higher for age group > 45 years compared to the other group (≤ 45 years) (Figs 4 and 5) and also significant differences are noted between rod thresholds in central and parafoveal locations. The former findings are in agreement with the previous studies that have studied rod sensitivity as a function of age [40–42, 71]. Rod sensitivity corrected for the expected absorption of light by the lens in the older group (mean age—70 years), show at least 0.4 log unit loss in sensitivity compared to the younger group (mean age—25 years) [41, 42]. Similarly, Jackson and Owsley showed that the loss in scotopic sensitivity is 0.08 log units per decade [40]. The loss in rod photoreceptors with ageing is well documented [20, 60, 61, 72], however, that may not fully account for the loss in rod sensitivity with ageing and differences in sensitivity between central and parafoveal locations. It is because the latter shows poor correlation with the magnitude of loss in rod density at different retinal eccentricities [41]. The neural basis for the increase in rod thresholds with ageing could be post-receptoral in origin, such as the steady loss of ganglion cells with increasing age [53, 55, 63]. The decrease in scotopic pattern ERG [73], dark-adapted scotopic b-wave [74] and VEP amplitudes associated with ageing [75], suggest the role of post-receptoral or post retinal circuitry in decreased contrast sensitivity with ageing. Also, the role of cortical neurons in the increase of rod FMT cannot be discounted as discussed earlier in the manuscript.

### Comparing rod and cone flicker modulation thresholds

Although not statistically significant, the rate of increase in central rod FMTs for the age group > 45 years was greater than central cone FMT (p = 0.15). This trend is consistent with the findings of Jackson et al. [40], who also showed the rate of increase in scotopic thresholds (-0.08 log units per decade) was greater than photopic thresholds (-0.04 log units per decade) with increasing age in normal healthy participants. However, it is be noted that Jackson et al. [40] used a linear model to describe their photopic and scotopic sensitivity data for the age range between ~ 20–80 years, contrary to the bilinear fit we used. The following potential differences between these studies may account for the discrepancies. The first major difference involves the state of background adaptation of rod photoreceptors. The earlier study measured rod thresholds when the retina was fully dark-adapted (40 minutes), whilst in the present study, we measured rod thresholds after adaptation to a uniform background of luminance (0.5 cd/m^2^) for a brief duration (90sec). The other differences include stimulus specifications such as briefly presented stimulus and not flickering stimuli, area of the retina (18°) tested compared to 5° (this study) and stimulus duration (200 ms vs 600 ms). The bilinear fit suggests that effects of ageing in a flicker test start to manifest after the age of 45 years unlike the functions such as photopic and scotopic flicker sensitivity measured using pulsed pedestal stimuli and mechanisms responsible for the detection of flicker detection and pulse stimuli are different [76]. However, we do see an agreement with a couple of studies with regards to the bilinear model, used to describe the photopic flicker thresholds as a function of age [30, 77]. Besides, approximately 89% (125/140) of participants in this study showed higher rod FMTs compared to cone FMTs. This finding is similar to Jackson et al. [40], who reported that 80% of the normal healthy participants had higher scotopic thresholds than photopic thresholds with increasing age. The finding of approximately similar rate of change for rod versus cone thresholds beyond 45 years of age (indicated by slopes in Fig 6), accompanied by markedly different slopes for each of these thresholds as a function of age (Fig 4) suggests mediation of two different mechanisms or photoreceptors classes that may be responsible for detection these stimuli.

We are aware of a couple of study limitations. One is that we had a limited number of participants > 70 years and other was that testing was done in an Indian population, whose average life expectancy is expected to be lower than that of North American and European population [78]. Therefore the results above 70 years of age rely largely on extrapolation. The results obtained in this study are more likely to reflect the true changes in flicker sensitivity because of the normal physiological ageing processes in the retina, as mentioned earlier in the manuscript. There is no obvious reason to assume that this ageing process would be different in the Indian population when compared to other populations.

## Conclusions

Cone-and rod-mediated FMTs remain relatively unchanged from 20 to about 45 years of age. Beyond this age, both rod and cone thresholds increase rapidly. The dataset obtained in this study describes the effects of normal ageing and will be a useful reference in the detection of rod and cone losses in preclinical disease, for the monitoring of disease progression and also help evaluate drug efficacy in patients with wet AMD, undergoing active treatment such as the use of the anti-vascular endothelial growth (VEGF) injections.
